# Hydrophilic Aldehyde-Functional Polymer Brushes: Synthesis, Characterization, and Potential Bioapplications

**DOI:** 10.1021/acs.macromol.2c02471

**Published:** 2023-02-22

**Authors:** Emma E. Brotherton, Edwin C. Johnson, Mark J. Smallridge, Deborah B. Hammond, Graham J. Leggett, Steven P. Armes

**Affiliations:** † Dainton Building, Department of Chemistry, The University of Sheffield, Brook Hill, Sheffield, South Yorkshire S3 7HF, U.K.; ‡ GEO Specialty Chemicals, Hythe, Southampton, Hampshire SO45 3ZG, U.K.

## Abstract

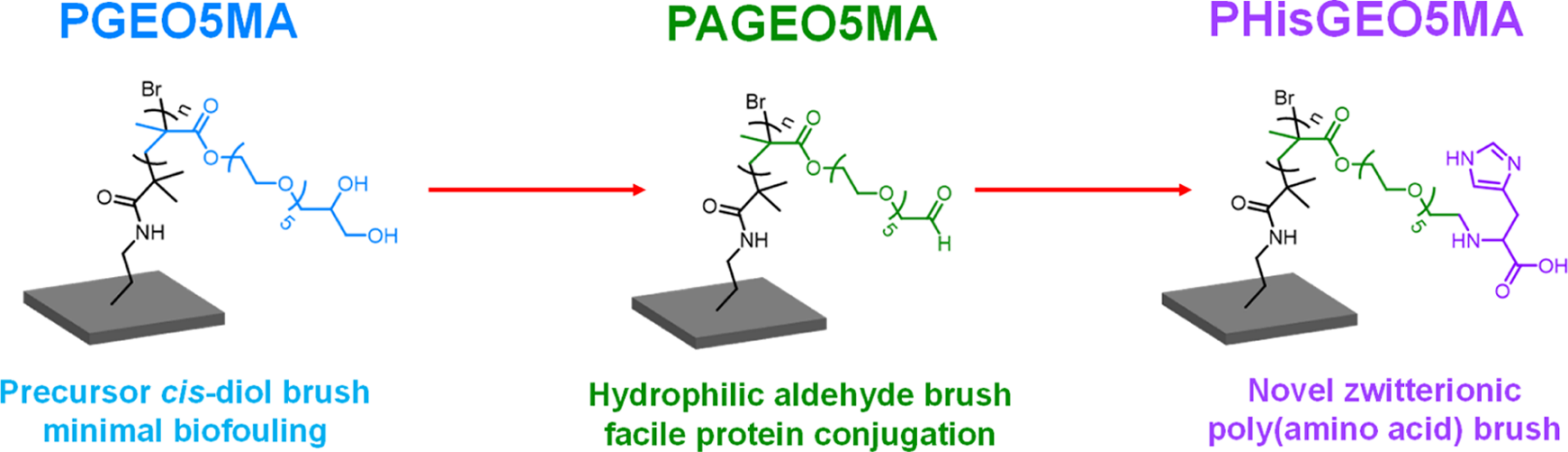

Surface-initiated activators regenerated by electron transfer atom transfer radical polymerization (ARGET ATRP) is used to polymerize a *cis*-diol-functional methacrylic monomer (herein denoted GEO5MA) from planar silicon wafers. Ellipsometry studies indicated dry brush thicknesses ranging from 40 to 120 nm. The hydrophilic PGEO5MA brush is then selectively oxidized using sodium periodate to produce an aldehyde-functional hydrophilic PAGEO5MA brush. This post-polymerization modification strategy provides access to significantly thicker brushes compared to those obtained by surface-initiated ARGET ATRP of the corresponding aldehyde-functional methacrylic monomer (AGEO5MA). The much slower brush growth achieved in the latter case is attributed to the relatively low aqueous solubility of the AGEO5MA monomer. X-ray photoelectron spectroscopy (XPS) analysis confirmed that precursor PGEO5MA brushes were essentially fully oxidized to the corresponding PAGEO5MA brushes within 30 min of exposure to a dilute aqueous solution of sodium periodate at 22 °C. PAGEO5MA brushes were then functionalized via Schiff base chemistry using an amino acid (histidine), followed by reductive amination with sodium cyanoborohydride. Subsequent XPS analysis indicated that the mean degree of histidine functionalization achieved under optimized conditions was approximately 81%. Moreover, an XPS depth profiling experiment confirmed that the histidine groups were uniformly distributed throughout the brush layer. Surface ζ potential measurements indicated a significant change in the electrophoretic behavior of the zwitterionic histidine-functionalized brush relative to that of the non-ionic PGEO5MA precursor brush. The former brush exhibited cationic character at low pH and anionic character at high pH, with an isoelectric point being observed at around pH 7. Finally, quartz crystal microbalance studies indicated minimal adsorption of a model globular protein (BSA) on a PGEO5MA brush-coated substrate, whereas strong protein adsorption via Schiff base chemistry occurred on a PAGEO5MA brush-coated substrate.

## Introduction

When polymer chains are tethered to a surface at a sufficiently high concentration such that they extend away from the surface, they are known as “polymer brushes”.^1,2^ Such systems have been extensively explored in the context of surface lubrication,^3−5^ the design of high performance anti-biofouling surfaces,^6−9^ the production of anti-bacterial surfaces,^10^ and as integral components of (bio)sensors.^11−15^ The development of copper-catalyzed atom transfer radical polymerization (ATRP) by Matyjaszewski and co-workers^16^ has stimulated this field by enabling the convenient synthesis of a wide range of polymer brushes of controllable thickness from a monolayer of surface initiator sites on a planar substrate using the so-called “grafting from” approach.^17^ Early studies involved hydrophobic brushes comprising poly(methyl methacrylate)^18^ or poly(*n*-butyl acrylate).^19^ However, various examples of hydrophilic brushes quickly became the focus of considerable attention, not least because they provide access to stimulus-responsive surfaces.^20^ Examples include thermoresponsive brushes based on poly(*N*-isopropyl acrylamide)^21−24^ or poly(sulfopropylbetaines)^25^ and pH-responsive brushes based on various tertiary amine methacrylates^26−29^ or poly(methacrylic acid).^30−32^

There have been various studies of the chemical derivatization of polymer brushes.^28,33−35^ For example, poly(2-hydroxyethyl methacrylate) brushes can be either esterified^36^ or oxidized to introduce desired functionality.^37^ Similarly, the pendent epoxy groups within poly(glycidyl methacrylate) brushes can be reacted with *n*-octylamine^38^ or *n*-propylamine^39^ and the tertiary amine groups in poly(2-dimethylamino)ethyl methacrylate can be quaternized using various alkyl halides.^33,40^ Zou et al. investigated the functionalization of periodate-oxidized poly[*N*-(2,3-dihydroxypropyl)acrylamide] (PDHPA) brushes with bovine serum albumin via reductive amination.^41^ However, brush derivatization protocols typically involve the use of organic solvents and often produce relatively low degrees of functionalization.

Recently, we reported the synthesis of a new hydrophilic methacrylic monomer, GEO5MA (see Scheme S1a).^42^ The pendent *cis*-diol group on this monomer can be selectively oxidized using sodium periodate to afford a rare example of an aldehyde-functional water-soluble monomer, AGEO5MA (see Scheme S1b). Alternatively, GEO5MA can be homopolymerized and the resulting PGEO5MA can be readily converted into PAGEO5MA by treatment with an aqueous solution of sodium periodate under mild conditions. Herein, we exploit this chemistry to prepare new examples of *hydrophilic* aldehyde-functional polymer brushes. According to the literature, such brushes are expected to be of considerable interest for various bio-applications.^37,43−46^ This is because they should enable facile conjugation of proteins or enzymes in aqueous solution at ambient temperature. Moreover, such brushes should be readily derivatized with an amino acid (e.g., histidine) to produce a new poly(amino acid methacrylate) brush via Schiff base chemistry. These two concepts are exemplified in the present study.

## Results and Discussion

**Figure 1:**
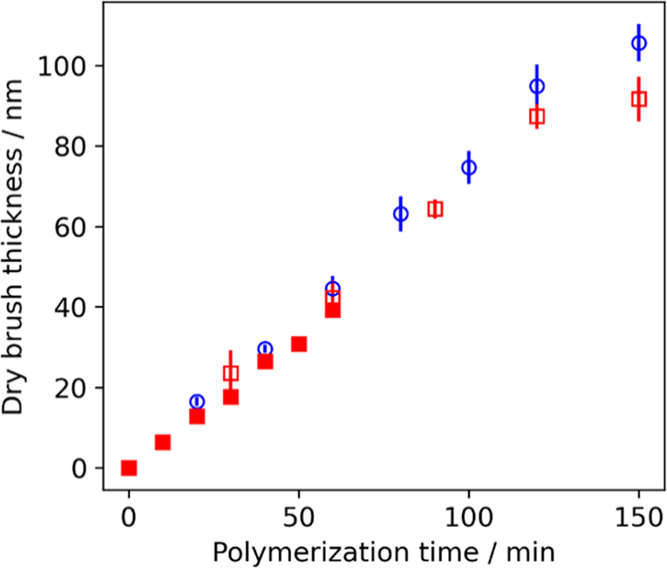
Evolution in dry brush thickness determined by ellipsometry during the SI-ARGET ATRP of GEO5MA at 22 °C using Protocol 1 (blue circles; individual initiator-functionalized wafers immersed within the same reaction solution in separate sample vials were periodically removed in turn) and Protocol 2 (red squares; a single initiator-functionalized silicon wafer was withdrawn periodically for characterization before being returned to the same reaction mixture. N.B. Open and filled red squares indicate data obtained for brush syntheses in which the wafer was removed at either 30 or 10 min intervals, respectively). Further formulation details are provided in the Experimental Section.

**Scheme 1:**
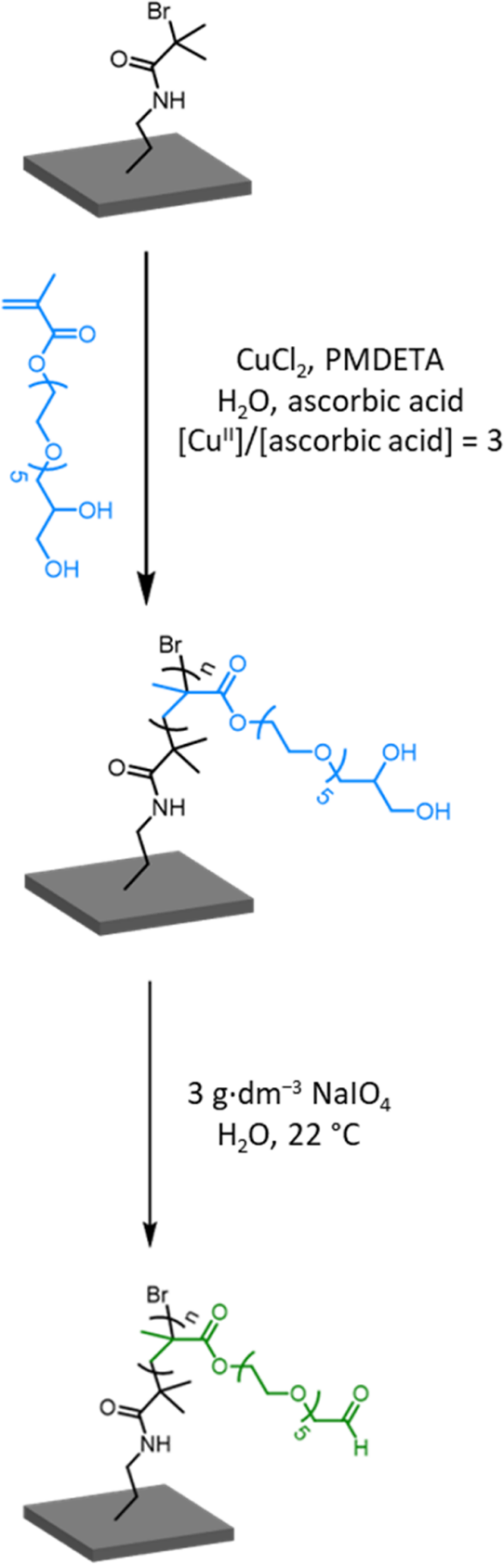
Reaction Scheme for the Synthesis of a PGEO5MA Brush via SI-ARGET ATRP of GEO5MA Followed by Periodate Oxidation Under Mild Conditions to Afford an Aldehyde-Functional PAGEO5MA Brush

PGEO5MA brushes were grown from a planar surface via surface-initiated activators regenerated by electron transfer atom transfer radical polymerization (SI-ARGET ATRP). More specifically, an aqueous CuCl_2_/*N*,*N*,*N′*,*N″*,*N″*-pentamethyldiethylenetriamine (PMDETA) catalyst was used to grow brushes from 3-(2-bromoisobutyramido)-propyl triethoxysilane (BiBB-APTES) coated silicon wafers at 22 °C using a GEO5MA concentration of 45% v/v and ascorbic acid as the reducing agent ([Cu(II)]/[ascorbic acid] molar ratio = 3), see Scheme 1. This surface ARGET ATRP protocol has been reported to yield a relatively high surface grafting density of 0.1 chains per nm^2^.^47−50^ The polymerization kinetics were monitored using two different synthesis protocols. Protocol 1 involved placing individual wafers in different reaction vessels and immersing each wafer in the same stock reaction solution. Each wafer was then removed from its vial at a different time point during the polymerization followed by copious rinsing (using ethanol and deionized water) and air-drying. Protocol 2 involved using one wafer in a single reaction vial and repeatedly (re)immersing the wafer in the reaction solution. During the ensuing polymerization, this wafer was periodically withdrawn, rinsed, and air-dried to enable its dry brush thickness to be determined by spectroscopic ellipsometry before being returned to the original reaction vial (Figure 1). Both protocols enable the polymerization kinetics to be monitored, which allows assessment of the pseudo-living character of the growing brush chains. In principle, a linear evolution in dry brush thickness over time indicates a well-controlled polymerization.^17,34,51^

Ellipsometry data were modeled using a single polymer Cauchy layer on native silicon dioxide with good fits being achieved in all cases (Figure S1). This indicates a relatively uniform brush thickness for each sample (with the interrogated surface area corresponding to around 50% of the total sample area). For Protocol 1, a highly linear increase in dry PGEO5MA brush thickness up to 95 nm was observed within 120 min at 22 °C, suggesting a well-controlled pseudo-living polymerization with minimal termination (open blue circles, Figure 1).^17,51^ However, deviation from linearity is observed for longer polymerization times, which suggests premature chain termination. Kinetic data were reported by Edmondson and co-workers for the growth of a closely related *cis*-diol-functional methacrylic polymer brush [i.e., poly(glycerol monomethacrylate), or PGMA].^29^ In this prior study, surface ATRP was conducted at ambient temperature using an anionic macroinitiator and a 1:1 v/v methanol/water mixture. PGMA brush growth was initially linear over the first 200 min, but slower kinetics and premature chain termination resulted in a dry PGMA brush thickness of only 17 nm after 21 h.

Protocol 2 produced comparable dry brush thicknesses to those obtained with Protocol 1. More specifically, a dry brush thickness of 88 nm was obtained within 120 min at 22 °C (red open squares, Figure 1). The linear nature of this plot suggests remarkably high re-initiation efficiency. A second experiment was performed using Protocol 2 over 60 min (red filled squares, Figure 1). These two data sets indicate good reproducibility for this surface-initiated ARGET ATRP formulation. Unfortunately, periodic removal/re-immersion of the silicon wafer eventually led to gelation of the reaction solution over longer time scales when using Protocol 2, which precluded further kinetic measurements with this method.

**Figure 2:**
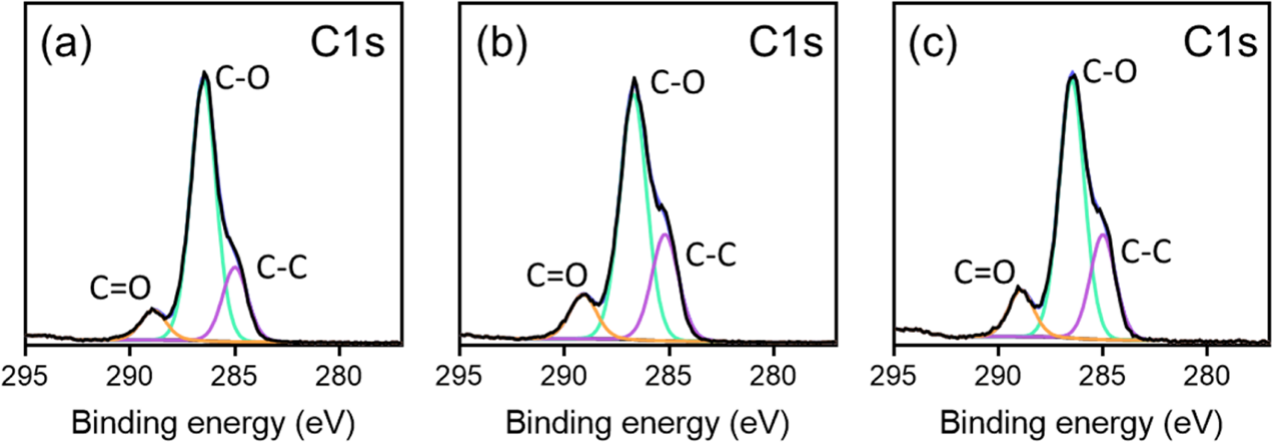
High-resolution C1s spectra obtained by XPS for (a) a PGEO5MA brush, (b) the corresponding periodate-oxidized PGEO5MA brush, and (c) a PAGEO5MA brush grown using AGEO5MA monomer.

X-ray photoelectron spectroscopy (XPS) was used to analyze the surface composition of an initiator-functionalized wafer and a PGEO5MA brush with a dry thickness of 97 nm (obtained using Protocol 1 after 120 min at 22 °C). Comparison of the high-resolution N1s and Br3d signals recorded for the initiator-functionalized wafer indicated a Br/N atomic ratio of ∼0.50, which suggests that approximately half of the primary amine groups on the initial APTES-treated wafer reacted with the 2-bromoisobutyryl bromide (Figure S2). Similar results were reported by Morse and co-workers for initiator-functionalized quartz fibers prepared by a similar protocol using the same reagents.^52^ Inspecting the survey spectra, the Si2s and Si2p signals corresponding to the underlying silicon wafer are clearly evident for the initiator-functionalized wafer but are absent for the PGEO5MA brush-coated wafer (Figure S3). A high-resolution C1s spectrum was acquired for the PGEO5MA brush (Figure 2a). The C1s signal was fitted using three components with binding energies of 285.0, 286.5, and 288.9 eV, which correspond to C–C, C–O, and O=C–O, respectively. The experimental atomic ratios for these components were 3.5:12:1.5, which is close to the theoretical atomic ratios of 3:13:1.

Selective oxidation of PGEO5MA brushes was achieved by immersion in an aqueous solution of sodium periodate at 22 °C to produce the corresponding hydrophilic aldehyde-functional PAGEO5MA brushes (Scheme 1). Recently, we reported that a periodate/*cis*-diol molar ratio of unity was required to achieve complete oxidation of the pendent *cis*-diol groups on a PGEO5MA homopolymer dissolved in aqueous solution.^42^ In contrast, oxidation of PGEO5MA brushes required a large excess of periodate owing to the relatively low mass of the grafted chains (estimated to be approximately 5 μg·cm^–2^). Zou et al. found that a 3.0 g·dm^–3^ aqueous solution of sodium periodate was sufficient to fully oxidize a *cis*-diol-functional PDHPA brush (dry brush thickness = 32 nm) within 60 min at ambient temperature,^41^ so similar conditions were employed in the present study. In the present case, the extent of oxidation of the PGEO5MA brush was monitored over time using ellipsometry (Figure S4) and XPS (Figure S5). PGEO5MA brushes (initial dry thickness = 74 to 120 nm) were immersed in turn into a 3.0 g dm^–3^ aqueous solution of sodium periodate for varying time periods at 22 °C prior to rinsing with deionized water and air-drying (Scheme 1). As expected, a monotonic reduction in dry brush thickness was observed by ellipsometry (Table S1 and Figure S4).^53^ The optimum oxidation time was empirically determined to be 30 min because this led to a reduction in dry brush thickness by approximately 8.5%,^53^ which corresponds to the loss of one formaldehyde per *cis*-diol repeat unit as the latter moiety is oxidized to produce a pendent aldehyde group (Scheme 1). However, longer reaction times led to further reduction in the brush thickness, which suggests some degree of brush degrafting. Nevertheless, we are confident that the rate of brush degrafting is appreciably slower than the rate of periodate oxidation of the *cis*-diol units to form aldehyde groups. This is supported by our observation that the dry brush remains relatively smooth after periodate oxidation for 30 min, with a significant increase in surface roughness only being observed over longer reaction times. Preliminary experiments confirmed that employing higher periodate concentrations also led to brush degradation (Figure S6). Indeed, significant brush degrafting was observed under harsher conditions (>0.5 M periodate for 24 h).

**Scheme 2:**
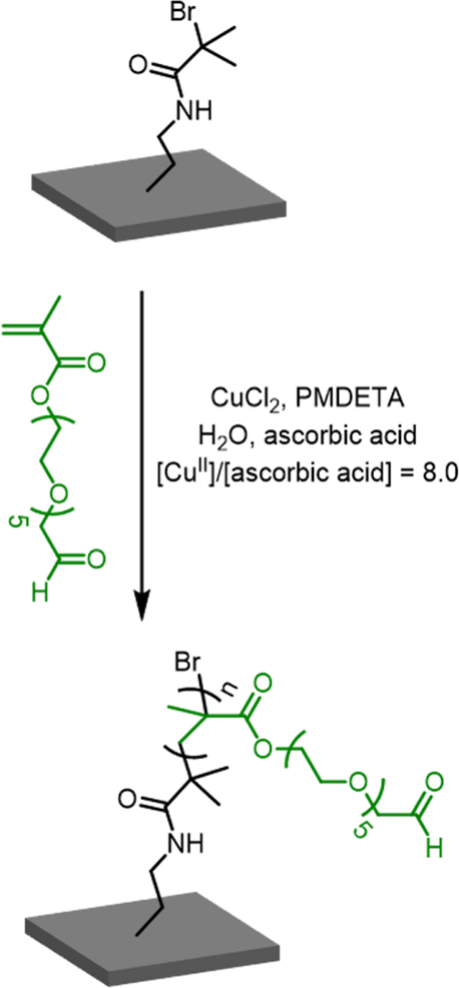
Reaction Scheme for the Direct Synthesis of a PAGEO5MA Brush via SI-ARGET ATRP of an Aldehyde-Functional Methacrylic Monomer (AGEOMA)

The extent of oxidation of the pendent *cis*-diol groups was confirmed by XPS. To provide a suitable reference material, a PAGEO5MA brush of 37 nm dry thickness was prepared by polymerizing AGEO5MA monomer (synthesized as reported by Brotherton et al.)^42^ from an initiator-functionalized silicon wafer via ARGET ATRP (Scheme 2). The C1s spectrum for the periodate-treated PGEO5MA brush is essentially identical to that recorded for the PAGEO5MA reference brush grown using the AGEO5MA monomer (Figure 2). Indeed, a C–C/C–O/C=O atomic ratio of approximately 4:10:2 was determined for the periodate-treated PGEO5MA brush, which is identical to the 4:10:2 atomic ratio obtained for the PAGEO5MA reference brush (Table 1). Both the PAGEO5MA and the periodate-oxidized PGEO5MA brush differ from the PGEO5MA brush. For comparison, the theoretical atomic ratio for a PAGEO5MA brush is 3:11:2. The carbonyl surface composition data summarized in Table 1 suggest a high degree of functionalization of (11.2−8.2)/(11.4−8.2) = 94%. The corresponding changes in the O1s core-line spectra are presented and briefly discussed in the Supporting Information (see Figure S7 and Table S2).^54^

**Table 1:** Summary of the High-Resolution C 1s Data Obtained by XPS Analysis of a PGEO5MA Brush, a Periodate-Oxidized PGEO5MA Brush, and a PAGEO5MA Brush (Synthesized Using AGEO5MA Monomer), Indicating the Relative Amounts of Each of the C–C, C–O, and C=O Components, Respectively

	XPS surface composition: atom %		
polymer brush	C–C	C–O	C=O
PGEO5MA	20.4	71.4	8.2
NaIO_4_-oxidized PGEO5MA	26.8	62.0	11.2
PAGEO5MA	25.6	63.0	11.4

In summary, the XPS and ellipsometry data indicate that essentially full oxidation of the *cis*-diol groups can be achieved within 30 min when using 3.0 g dm^–3^ sodium periodate at 22 °C. Furthermore, the extent of brush degrafting that occurs under such mild conditions appears to be negligible (Figure S4). Importantly, this is significantly higher than the degree of aldehyde functionality of approximately 49% reported by Klok et al., who used Albright-Goldman oxidation to derivatize a poly(2-hydroxyethyl methacrylate) (PHEMA) brush in DMSO.^37^ Moreover, this prior route to aldehyde-functional brushes produced relatively hydrophobic brushes, unlike the hydrophilic brushes reported herein. Clearly, the wholly aqueous derivatization protocol described herein should be highly attractive for potential bio-applications.

**Scheme 3:**
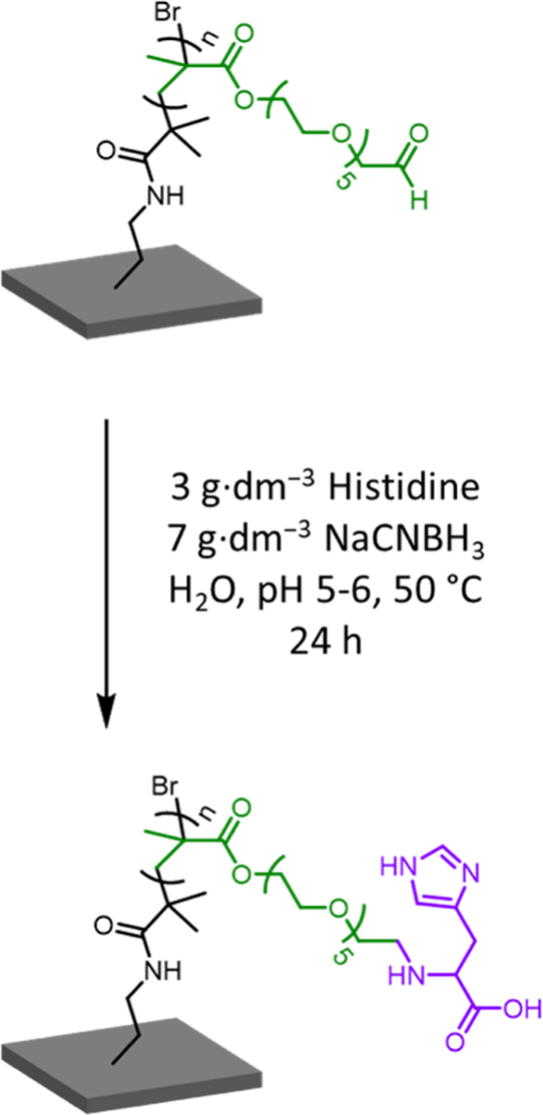
Reaction Scheme for the Functionalization of PAGEO5MA With Histidine via Reductive Amination at 50 °C

PAGEO5MA brushes prepared via periodate oxidation were subsequently reacted with histidine via Schiff base chemistry, followed by reductive amination using NaCNBH_3_ ([NaCNBH_3_] = 7.0 g·dm^–3^), see Scheme 3. This amino acid was selected because its successful conjugation was expected to confer pH-dependent zwitterionic character following reductive amination.^55^ Following our recently reported protocol for the derivatization of an aqueous dispersion of PAGEO5MA_26_-stabilized nanoparticles with histidine,^55^ the initial Schiff base reaction and the subsequent reductive amination was allowed to proceed for 24 h at 50 °C using a one-pot protocol (see Scheme 3).

Dry brush thicknesses of 82 and 109 nm were determined by ellipsometry for a periodate-oxidized PGEO5MA brush and the corresponding histidine-functionalized PGEO5MA brush (PHisGEO5MA), respectively. Given that the dry brush thickness is proportional to the molecular weight of the repeat units,^53^ the increase in dry brush thickness can be used to estimate the mean degree of histidine functionalization.^38^ For full histidine conjugation, the molecular weight of the repeat units should increase from 351 to 490 g mol^–1^, which would result in a theoretical 40% increase in dry brush thickness. In practice, a 32% increase in dry brush thickness is observed. Hence, the mean degree of histidine functionalization of the periodate-oxidized PGEO5MA brush can be estimated to be 32 ÷ 40 = 0.80 (or 80%) from ellipsometry measurements.

**Figure 3:**
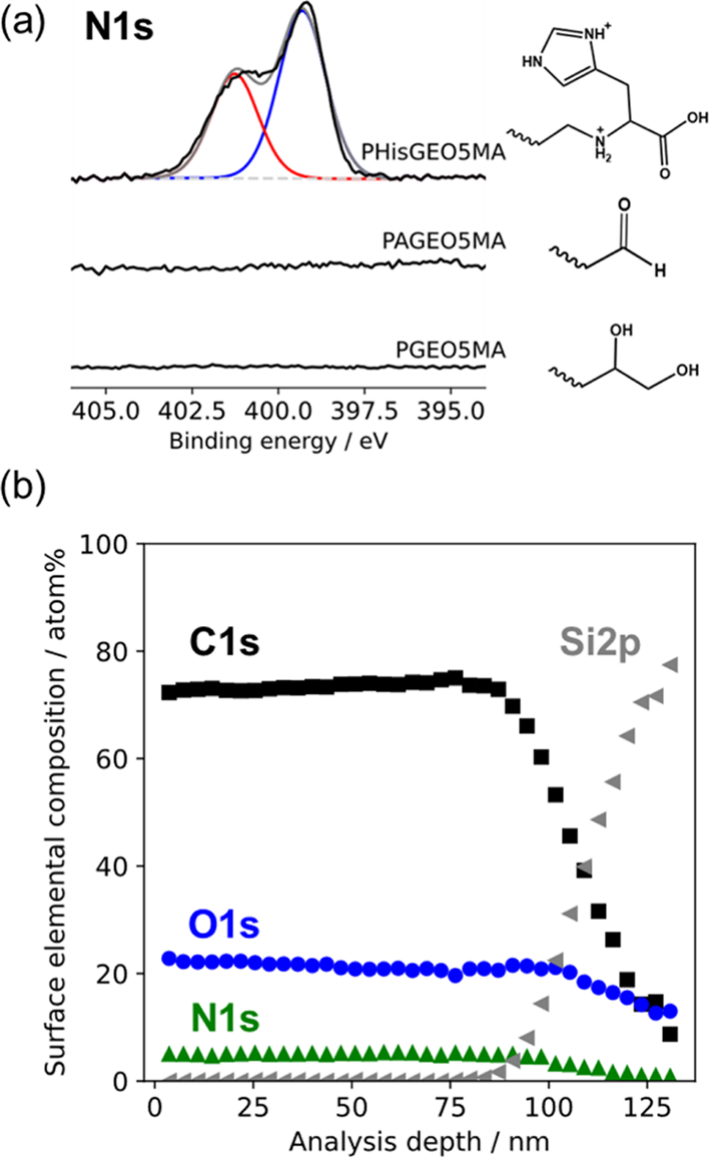
(A) High-resolution N1s spectra recorded for a 97 nm PGEO5MA brush, a 90 nm periodate-oxidized PGEO5MA brush, and a 99 nm PHisGEO5MA brush. The latter brush was exposed to an aqueous solution of 0.01 M HCl (pH 2) prior to drying for XPS analysis. The PHisGEO5MA spectrum can be satisfactorily fitted using two components, which correspond to the aliphatic secondary amine nitrogen atom (red) and the two aromatic imidazole nitrogen atoms (blue), respectively. (B) Elemental composition of a PHisGEO5MA brush as a function of analysis depth as determined by XPS depth profiling. Representative spectra are shown in Figure S8.

Inspecting the high-resolution N1s spectra recorded for each brush provides further information (Figure 3). As expected, no N1s signal is observed for either the 97 nm PGEO5MA precursor brush or the 90 nm periodate-oxidized PGEO5MA brush (Figure 3). These dry brush thicknesses are much greater than the maximum XPS sampling depth of 10 nm,^56^ so the amide-based ATRP initiator (and any unreacted APTES) is not discernible. In contrast, a strong N1s signal is observed for the PHisGEO5MA brush (Figure 3), indicating successful histidine conjugation.

The degree of histidine functionalization was calculated from the N/O atomic ratios determined by XPS. Comparing the experimental N/O atomic ratio to its maximum theoretical value for 100% functionalization indicated a mean degree of functionalization of 81% for the PHisGEO5MA brush, which is consistent with that calculated from the increase in brush thickness determined by ellipsometry. At first sight, this is roughly equal to that reported by Bilgic and Klok, who achieved degrees of functionalization of up to 79% for oxidized PHEMA brushes reacted with benzylamine, as calculated using N/C atomic ratios.^37^ However, given that only ∼49% of the PHEMA brush was oxidized to the corresponding aldehyde groups, this suggests an overall degree of functionalization of ∼39% for this prior study, which also required the use of a noxious organic solvent (DMSO). On the other hand, the degree of functionalization of such PAGEO5MA brushes is less than that achieved for soluble PAGEO5MA chains in aqueous solution, for which more than 98% functionalization was achieved using several amino acids (including histidine).^42,55^ Presumably, the lower reactivity of the brush system simply reflects the greater steric congestion of such surface-confined chains.^57^

The comparable mean degrees of brush functionalization calculated from the ellipsometry and XPS data suggest that histidine functionalization (and thus PGEO5MA oxidation) occurs throughout the entire brush layer. However, the maximum XPS sampling depth of 10 nm is much less than the mean brush thickness.^56^ Thus XPS depth profiling experiments were conducted to determine whether the histidine groups—which are the sole source of nitrogen atoms—are indeed uniformly distributed throughout the brush layer. It is well-known that polymers exhibit high rates of degradation during surface etching via ion bombardment, which can dramatically reduce depth resolution.^58−61^ Fortunately, this problem can be addressed by employing a cluster ion source for depth-profiling studies.^58−61^ Such sources provide excellent control over the etching process. For example, XPS depth profiling studies of poly(glycidyl methacrylate) (PGlyMA) and GlyMA copolymer brushes have been reported using C_60_^+^ or coronene (C_24_H_12_^+^) sources, respectively.^62,63^ The recent development of giant gas cluster sources provides even finer control over surface etching by facilitating the selective removal of contaminants from polymer surfaces.^64,65^ Herein, we used an Ar_3000_^+^ ion source to perform an XPS depth-profiling experiment on a PHisGEO5MA brush. The resulting XPS data are shown in Figure 3b (and Figure S8) for a dry brush thickness of 109 nm and a mean degree of histidine functionalization of approximately 80%. The N1s signal assigned to the pendent histidine groups is approximately 5 atom % within the upper brush surface and remains essentially constant as the brush layer is gradually ablated. Eventually, the underlying silicon wafer is reached at a depth of approximately 109 nm, as indicated by the pronounced upturn in the Si2p signal and the corresponding drop-off in the N1s and C1s signals. Hence this depth profiling study provides strong evidence for uniform histidine functionalization throughout the brush layer.

**Figure 4:**
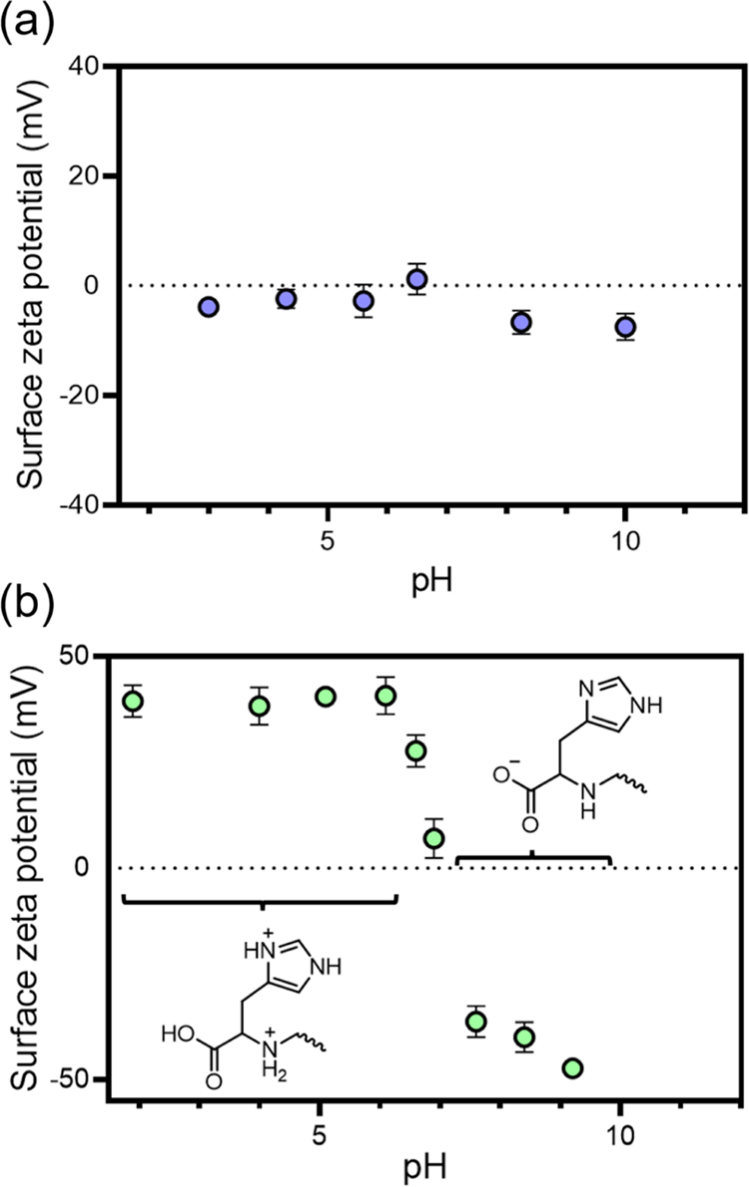
Surface zeta potential (ζ) potential vs pH curves recorded for (a) a PGEO5MA brush of 97 nm dry thickness and (b) a PHisGEO5MA brush of 99 nm dry thickness (degree of histidine functionalization = 81% as judged by XPS).

Owing to its dual carboxylic acid and amine functionality, histidine exhibits pH-dependent zwitterionic character in aqueous solution. Thus, functionalization of the non-ionic PAGEO5MA brush with this amino acid should produce a significant change in its electrophoretic behavior. In a related study, we reported that adjusting the solution pH leads to a substantial change in the surface ζ potential of a zwitterionic poly(cysteine methacrylate) brush.^66^ Accordingly, surface ζ potential studies were conducted to characterize the PHisGEO5MA brush obtained after periodate oxidation, histidine conjugation, and reductive amination (Scheme 3). Surface ζ potentials were recorded using a Malvern Nanosizer instrument equipped with a Malvern Surface Zeta Potential ZEN1020 dip cell. In essence, ζ potentials are determined for suitable tracer nanoparticles (see the Experimental Section for further details) at varying distances from the surface of interest. For cationic surfaces, cationic tracer nanoparticles were used to ensure that no nanoparticle adsorption occurred. Similarly, non-ionic tracer nanoparticles were used to characterize either neutral or anionic surfaces.^66,67^ Monitoring the change in the apparent ζ potential of the tracer nanoparticles enables the surface ζ potential of each brush to be determined at a given pH (Figure S9). As expected, the surface ζ potential of a 97 nm PGEO5MA brush remained approximately neutral over a wide range of solution pH (Figure 4a).

For a corresponding 99 nm PHisGEO5MA brush, strongly positive ζ potentials are observed at low pH owing to protonation of the imidazole ring, the secondary amine linkage and the pendent carboxylic acid group on each histidine repeat unit (Figure 4b). Moreover, similarly negative ζ potentials are observed at high pH owing to ionization of the carboxylic acid groups and deprotonation of the imidazole rings and/or secondary amine linkages. An isoelectric point (corresponding to zero net charge on the brush chains) is observed at around pH 7.0. As a comparison, we recently reported aqueous electrophoretic data for PHisGEO5MA_26_-stabilized vesicles in 1 mM KCl.^55^ In this case, an isoelectric point was obtained at pH 6.5 and comparable positive and negative ζ potentials were observed at low and high pH, respectively. It is perhaps worth emphasizing that substantial changes in the surface ζ potential can be achieved despite incomplete brush functionalization. This highlights the potential for PAGEO5MA brushes to act as hydrophilic scaffolds to which amine-functional molecules (e.g., dyes) can be readily conjugated. This concept will be explored in the near future.

Recently, we reported that PAGEO5MA-functionalized diblock copolymer worm gels exhibit strong mucoadhesive behavior.^68^ This is because the pendent aldehyde groups can react with the primary amine groups that are located at the surface of porcine urinary bladder mucosa.^68^ Conversely, the corresponding PGEO5MA-functionalized diblock copolymer worm gels exhibit minimal mucoadhesion. In view of these observations, quartz crystal microbalance (QCM) experiments were performed to determine the extent to which a model globular protein (bovine serum albumin, BSA) interacts with (i) a PGEO5MA brush and (ii) the corresponding PAGEO5MA brush. In principle, the former hydroxyl-rich brush system should be protein-repellent, whereas the latter aldehyde-functional brush system should be protein-adherent via Schiff base chemistry.

QCM is an established analytical technique that has been widely used to either assess the extent of protein adsorption onto polymer brushes or examine their anti-biofouling performance.^69−74^ In QCM measurements, adsorption modifies the resonant frequency of the quartz crystal sensor. This change in frequency, Δ*f*, is proportional to the mass of adsorbed material, *m*. The simplest model relating Δ*f* to *m* is the Sauerbrey equation,^75^ which is often used to calculate the mass of adsorbed protein.^69,73,76^

**Figure 5:**
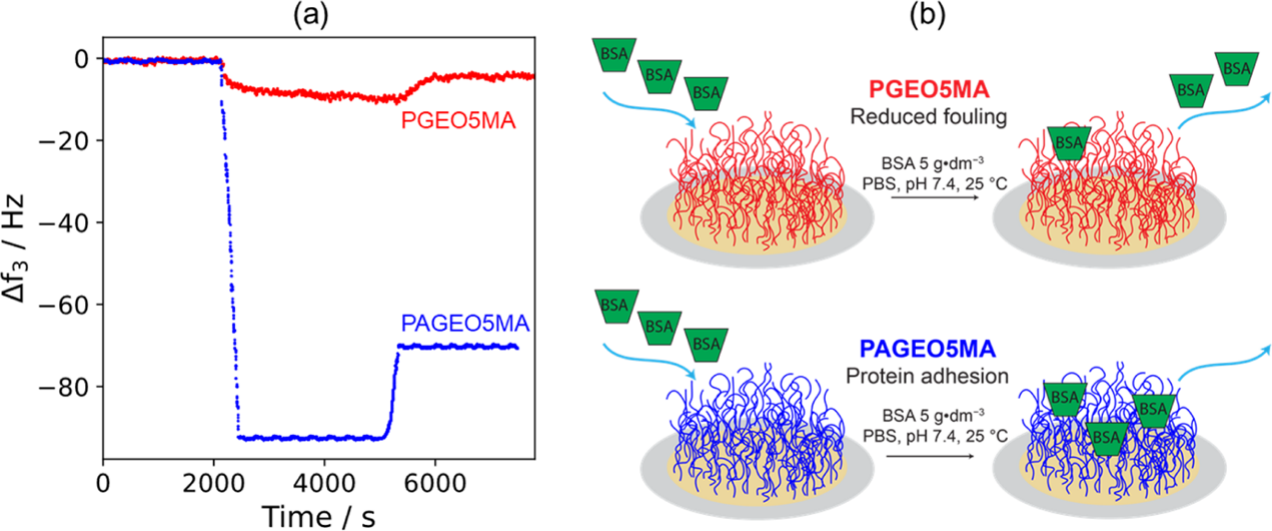
(a) Change in frequency, Δ*f*, observed over time for a silica QCM sensor coated with either a 15 nm PGEO5MA brush (red) or a 13 nm PAGEO5MA brush (blue) after exposure to an aqueous solution of BSA (5.0 g·dm^–3^) in PBS at pH 7.4. Using the Sauerbery equation, the corresponding adsorbed amount, Γ, was calculated to be 0.2 mg·m^–2^ for PGEO5MA and 4.3 mg·m^–2^ for PAGEO5MA. (b) Schematic cartoon illustrating the marked difference in behavior observed for anti-biofouling PGEO5MA brushes and protein-reactive PAGEO5MA brushes on exposure to BSA.

Figure 5 shows Δ*f* data observed for a PGEO5MA brush and a periodate-treated PGEO5MA (i.e., PAGEO5MA) brush when such systems are exposed in turn to an aqueous BSA solution in phosphate-buffered saline (PBS) buffer. BSA is commonly used as an exemplar protein for anti-biofouling experiments owing to its extensive characterization and low cost. The PBS buffer pH of 7.4 is above the isoelectric point for BSA, so this protein has anionic character under the experimental conditions.^77^ Relatively thin brushes (15 nm for PGEO5MA and 13 nm for PAGEO5MA) were selected for these experiments to minimize signal loss (so-called “hearing loss”) owing to dampening of the acoustic signal of the oscillator.^76^ XPS and ellipsometry studies confirmed that relatively thick, uniform PGEO5MA and PAGEO5MA brushes can be grown from planar silica substrates. Thus, any observed difference in BSA adsorption between such brushes can be solely attributed to the introduction of pendent aldehyde groups via periodate oxidation.

A reduction in frequency is observed on addition of the BSA solution for both the PGEO5MA and PAGEOMA brush. However, a much greater reduction is observed in the latter case. After rinsing both brushes with PBS buffer, an increase in frequency occurs as weakly adsorbed protein is removed. For the hydroxyl-rich PGEO5MA brush, the final frequency lies close to the original baseline. There is only a small change in Δ*f*, which corresponds to an adsorbed amount of just 0.2 mg·m^–2^. This suggests a very weak interaction between this brush and BSA. In contrast, the PAGEO5MA brush exhibits a much greater Δ*f*, which corresponds to an adsorbed amount of 4.3 mg·m^–2^. Clearly, this aldehyde-functional brush interacts strongly with the primary amine groups present at the surface of BSA via Schiff base chemistry. In principle, the resulting imine bonds are susceptible to hydrolysis but in practice, the formation of multiple imine bonds per protein should be sufficient to ensure permanent adsorption of this analyte via dynamic covalent chemistry. We envisage that the ability to switch between an anti-biofouling PGEO5MA brush and a PAGEO5MA brush that is capable of strong protein adhesion using a simple aqueous treatment under mild conditions may be advantageous for potential bio-applications. For example, it should be feasible to design a wide range of enzyme-conjugated brush systems.

## Conclusions

We report the synthesis of new aldehyde-functional hydrophilic polymer brushes using surface-initiated ARGET ATRP to polymerize GEO5MA from a planar silicon wafer followed by selective oxidation of the pendent *cis*-diol groups using a dilute aqueous solution of sodium periodate at 22 °C. By comparing to a reference brush prepared using an analogous aldehyde-functional methacrylic monomer (AGEOMA), XPS analysis confirmed that the degree of aldehyde functionalization of such PAGEO5MA brushes was at least 94% within 30 min of their exposure to sodium periodate. One such PAGEO5MA brush was subsequently functionalized via Schiff base chemistry using excess histidine, followed by reductive amination with sodium cyanoborohydride. By comparing N/O atomic ratios, XPS analysis indicated that the mean degree of histidine functionalization achieved for this wholly aqueous brush derivatization protocol was approximately 81% under optimized conditions. Moreover, XPS depth profiling confirmed a uniform concentration of histidine groups throughout the brush layer. Surface ζ potential measurements indicated that the resulting zwitterionic PHisGEO5MA brush exhibited cationic character at low pH and anionic character at high pH, with an isoelectric point observed at around pH 7. In contrast, the non-ionic precursor PAGEO5MA brush exhibited a near-zero surface ζ potential over the same pH range. Finally, QCM experiments confirm that a PGEO5MA brush is anti-biofouling, whereas the corresponding PAGEO5MA brush is strongly protein-adherent when challenged with a model globular protein (BSA). This is because the aldehyde groups on the latter brush can react with the primary amine groups of the protein to form multiple imine bonds. This suggests that such brushes could be decorated with a wide range of proteins, including enzymes.

## Experimental Section

### Materials

All reagents were used as received unless otherwise stated. GEO5MA monomer was synthesized by Dr C. Jesson at GEO Speciality Chemicals (Hythe, UK) and was used without further purification.^42^ (3-Aminopropyl)triethoxysilane (APTES; 99%), 2-bromoisobutyryl bromide (BiBB; 98%), sodium periodate (NaIO_4_; >99%), histidine (≥98%), sodium cyanoborohydride (NaCNBH_3_; 95%), 2,2,2-trifluoroethylamine (TFEA; 99.5%), and 1,4 dioxane were all purchased from Sigma-Aldrich (UK). Tetrahydrofuran (THF) and *N*,*N*,*N′*,*N″*,*N″*-pentamethyldiethylenetriamine (PMDETA; 98%) were purchased from Fisher Scientific (UK). Copper(II) chloride (CuCl_2_; 99%) was purchased from Acros Organics (UK). Test grade silicon wafers (100) were purchased from PI-KEM (Tamworth, UK). Deionized water was used for all experiments involving aqueous solutions.

### Methods

#### Spectroscopic Ellipsometry

Measurements were performed in air at 20 °C on bare planar silicon wafers, initiator-functionalized silicon wafers or polymer brush-functionalized silicon wafers using a J. A. Woollam M2000 V ellipsometer at a fixed angle of incidence of 75° normal to the sample surface. A wavelength range of 370–1000 nm was used to obtain two ellipsometry parameters (Ψ and Δ). These parameters were fitted to a two-layer model consisting of a native oxide layer and a Cauchy layer (Equation 1).
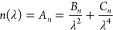
1

Data analysis and modeling were performed using Woollam CompleteEase software, which fits the Ψ and Δ values calculated using this two-layer model to the experimental data. The following Cauchy parameters were used: *A*_*n*_ = 1.4615, *B*_*n*_ = 0.00514 μm^–2^, and C_*n*_ = 0. The ellipsometer setup allowed a relatively large sampling area of approximately 0.5 cm × 1 cm, which corresponds to around 50% of the total area of each brush sample.

#### Surface ζ Potential Measurements via Laser Doppler Electrophoresis

Surface ζ potentials were calculated for selected polymer brushes from laser Doppler electrophoresis data obtained using Malvern Zetasizer instrument equipped with a Malvern Surface Zeta Potential ZEN1020 dip cell. Polymer brushes grown from planar silicon wafers (4 mm × 5 mm) were attached to the sample holder using an ethyl cyanoacrylate-based adhesive (Gorilla Super Glue, Gorilla Glue Europe A/S) and the wafer-loaded sample holder was placed into the Malvern ZEN1020 dip cell. The Zetasizer instrument setup detects forward-scattered light at an angle of 13° with the attenuator adjusted to 100% laser transmission (position eleven). Voltage selection was set to automatic (typically 10 V). The dip cell was placed in a cuvette containing 1.0 mL of either 0.003% w/w neutral PGMA_58_-PBzMA_500_ or cationic PMETAC_47_-PBzMA_100_ tracer nanoparticles [where PBzMA and PMETAC denote poly(benzyl methacrylate) and poly(2-(methacryloyloxy)ethyl trimethylammonium chloride), respectively] in the presence of 1 mM KCl at 25 °C. This nanoparticle concentration was chosen to provide an optimal derived count rate of 500 kcps under the stated operating conditions.^78^ Five slow-field reversal measurements were performed at four distances from the sample surface (125, 250, 375, and 500 μm), with each measurement comprising 15 sub-runs and a 1 min interval being allowed between measurements. Then three fast-field reversal measurements were performed at a distance of 1000 μm from the sample surface to calculate the electro-osmotic mobility of the tracer nanoparticles. In this case, each measurement consisted of 100 sub-runs with an interval of 20 s being allowed between each measurement. ζ potentials were calculated via the Henry equation using the Smoluchowski approximation.

#### X-ray Photoelectron Spectroscopy

Polymer brushes grown from planar silicon wafers were analyzed using a Kratos Axis Supra X-ray photoelectron spectrometer. Step sizes of 0.50 and 0.05 eV were used to record survey spectra and high-resolution spectra, respectively. In each case, spectra were recorded from at least two separate areas. The XPS data were analyzed using Casa XPS software (UK). All binding energies were calibrated with respect to the C1s saturated hydrocarbon peak at 285.0 eV.

#### XPS Depth Profiling

These experiments were conducted using a Kratos Supra instrument equipped with a monochromated aluminum source and an argon cluster source. First, spectra were recorded prior to surface etching. Then, surface etching was conducted using the argon cluster source (Ar3000+ clusters at 10 keV; ion beam current = 9.5 nA) for a predetermined time period and new spectra were recorded prior to further surface etching. This etching/analysis cycle was repeated until the C1s and N1s signals disappeared, which indicated that the entire brush layer had been etched. The cluster source was rastered over a 2 mm by 2 mm area to produce an etch crater and X-rays were collected from an area of 110 μm diameter at the center of each crater (X-ray emission current = 25 mA at 15 kV). High-resolution scans were recorded for O1s (one 30 s sweep), N1s (four 60 s sweeps), C1s (four 60 s sweeps), and Si2p (one 60 s sweep). All data were collected at a pass energy of 40 eV. Charge neutralization was used throughout at 0.4 A. A transmission function characteristic of the instrument was used for calibration to produce instrument-independent data, which were quantified using theoretical Schofield relative sensitivity factors modified to account for instrument geometry, any variation in penetration depth with energy and the angular distribution of the photoelectrons. High-resolution spectra were calibrated by assigning the C–C/C–H environment within the C1s signal to be 285.0 eV.

#### Quartz Crystal Microbalance Measurements

Quartz crystal microbalance sensors coated with a 50 nm silica overlayer (QSX 303, ∼5 MHz fundamental frequency) were purchased from Q-Sense (Sweden). Each sensor was cleaned according to the manufacturer’s instructions. This protocol involved (i) UV/O_3_ treatment for 15 min (Bioforce UV/O_3_ cleaner, ∼9 mW cm^–2^, λ = 254 nm), (ii) exposure to 2% w/w sodium dodecylsulfate solution for 30 min, (iii) copious rinsing with deionized water and drying under N_2_, and (iv) a final UV/O_3_ treatment for 15 min. The resulting sensors were then (i) amine-functionalized with APTES and (ii) initiator-functionalized with BiBB before (iii) brush growth using Protocol I described above. The dry brush thickness of a second wafer present in the reaction mixture during polymerization was determined by ellipsometry. This value was used to infer the thickness of the brush grown on the QCM sensor.

QCM measurements were performed using an openQCM NEXT instrument (Novatech Srl., Italy) equipped with a temperature-controlled cell connected to a Masterflex Digital Miniflex peristaltic pump (Cole-Parmer Instrument Company, UK). All experiments were conducted using PBS buffer (pH 7.4) and were not commenced until the sensor frequency exhibited a drift of less than 0.1 Hz·min^–1^; this typically occurred within an hour of filling the cell. Once a stable signal was obtained, a 5.0 g·dm^–3^ solution of BSA in PBS was passed through the cell at a flow rate of 0.025 mL·min^–1^ (minimum flow volume = 2.0 mL).

The adsorbed amount can be calculated using various models.^74,76,79^ The simplest and most widely applied model uses the Sauerbrey equation, which relates the change in frequency, Δ*f*, to the change in adsorbed mass per unit area, *m*
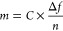
where *C* is a sensitivity constant [−0.177 (mg·m^–2^) × Hz^–1^], Δ*f* is the change in resonant frequency (Hz), and *n* is the overtone number. The third harmonic (*n* = 3) was used to calculate the adsorbed amount to avoid experimental artifacts associated with the fundamental harmonic that may occur if the sample mounting on the sensor is imperfect.^74,76,80^

### Synthesis Details

#### Preparation of Initiator-Functionalized Silicon Wafers

Silicon (100) wafers were cut into small pieces (∼1 × 1 cm^3^) before being UV–ozone cleaned for 60 min at 10^3^ Pa using a Bioforce Nanosciences ProCleaner. These wafers were then placed in test tubes along with a 3 mL glass sample vial containing ∼100 μL of APTES and the test tubes were sealed with a rubber septum before being placed in a 100 °C oven for 60 min. The resulting APTES-functionalized silicon wafers were removed from the oven and excess APTES was allowed to evaporate before washing the wafers with THF and drying them under a stream of compressed air. The wafers were then functionalized by immersion in a 0.1 M BiBB solution in 1,4-dioxane for 18 h at 22 °C. Finally, the wafers were rinsed extensively with 1,4-dioxane and water before drying under a stream of compressed air.

#### Polymerization Kinetics Experiments

SI-ARGET ATRP was used to grow PGEO5MA brushes from initiator-functionalized silicon wafers at an aqueous GEO5MA concentration of 45% v/v using GEO5MA/Cu(II)Cl_2_/PMDETA/ascorbic acid molar ratios of 1000:1:5:3 using one of the following two protocols.

##### Protocol 1

The catalyst, ligand, monomer, and water were weighed in turn into a 50 mL round-bottom flask containing a magnetic flea. The resulting solution was stirred for 10 min prior to addition of the ascorbic acid. The reaction mixture was stirred for a further 10 min to ensure formation of the active catalyst. Each initiator-functionalized silicon wafer was placed in a sealable 1.5 mL vial before being filled with the reaction mixture such that the volume of air remaining in each sealed vial was less than 0.1 cm^3^. Each wafer was removed from the reaction mixture after the desired polymerization time and rinsed extensively with ethanol and deionized water prior to drying under a stream of compressed air for ellipsometry studies.

##### Protocol 2

The catalyst, ligand, monomer, and water were pipetted into a 7 mL sample vial. The reaction mixture was stirred for 10 min followed by the addition of ascorbic acid. The polymerization mixture was then stirred for an additional 10 min. An initiator-functionalized silicon wafer was placed in the sample vial. The volume of air remaining in the vial was approximately 1 cm^3^. After 10 min, the wafer was removed from the reaction mixture, rinsed extensively with deionized water, and dried using a stream of compressed air. The dry brush thickness was determined by ellipsometry and the wafer was reimmersed in the reaction mixture. This protocol was repeated five times over a total “brush immersion” reaction time of 60 min.

The kinetics of surface-initiated polymerizations differ from that for the analogous solution polymerization, which makes a direct comparison somewhat problematic.^81,82^ Moreover, determination of the molecular weight of the brush chains via degrafting is not feasible for the planar silicon wafers employed in this study owing to the very small mass densities of grafted polymer (estimated to be 5 μg·cm^–2^). Thus, the brush grafting density is simply assumed to be comparable to brushes prepared using similar synthesis protocols.^48,49,83^

### Selective Oxidation of PGEO5MA Brushes Using Sodium Periodate

PGEO5MA brush-functionalized planar silicon wafers were immersed in a 3.0 g·dm^–3^ aqueous solution of sodium periodate for 30 min at 22 °C. Each wafer was rinsed extensively with deionized water and then dried using a stream of compressed air.

### Synthesis of the PAGEO5MA Reference Brush by SI-ARGET ATRP

A PAGEO5MA reference brush was prepared at an AGEO5MA concentration of 15% v/v in the presence of ascorbic acid according to the following protocol. AGEO5MA (0.87 mL, 3.1 mmol), water (4.79 mL), Cu(II)Cl_2_ (0.92 mg, 6.84 μmol), and PMDETA (50 μL) were added to a 7 mL sample vial. This reaction solution was stirred for 2 min to ensure thorough mixing before the addition of the ascorbic acid (0.15 mg, 0.85 μmol, 0.42 mM) and immersion of the silicon wafer. Each sample vial contained approximately 1 cm^3^ of air and the SI-ARGET ATRP of GEO5MA was allowed to proceed for 1–2 h at 22 °C. Each polymerization was quenched by removing the silicon wafer from the reaction mixture. Each wafer was rinsed extensively with deionized water and then dried using a stream of compressed air.

### Functionalization of PAGEO5MA Brushes with Histidine Followed by In Situ Reductive Amination

An aqueous solution containing 3 g·dm^–3^ histidine and 7 g·dm^–3^ NaCNBH_3_ was adjusted to pH 5–6. PAGEO5MA brush-functionalized silicon wafers were immersed in this aqueous solution for 24 h at 50 °C. Each wafer was removed from the reaction solution, rinsed extensively with deionized water, and then dried under a stream of compressed air.

## Supporting Information Available

Reaction scheme for GEO5MA monomer synthesis; ellipsometry data for four PGEO5MA brushes, XPS data for ATRP initiator-coated wafer; XPS survey spectra for an initiator-functionalized silicon wafer, a PGEO5MA brush, and a PAGEO5MA brush; ellipsometry data for a periodate-oxidized PGEO5MA brush; XPS C1s core-line spectrum for a periodate-oxidized PGEO5MA brush; reduction in dry brush thickness observed for a periodate-oxidized PGEO5MA brush over time; ellipsometry data for six PGEO5MA brushes and periodate-oxidized PGEO5MA brushes; XPS O1s core-line spectra for a PGEO5MA brush, periodate-treated PGEO5MA brush; summary table of assigned components (atom %) for O1s spectra for the same brushes; C1s, N1s, and Si2p core-line spectra recorded as a function of analysis depth for a PHisGEO5MA brush determined during an XPS depth profiling experiment; and raw data for surface ζ potential measurements on a PGEO5MA brush and a PHisGEO5MA brush (PDF)
