# Supplementary material for: Hydrophilic Aldehyde-Functional Polymer Brushes: Synthesis, Characterization, and Potential Bioapplications
Source: Macromolecules. 2023 Feb 22;56(5):2070–80. doi: 10.1021/acs.macromol.2c02471 (PMC10018759; doi:10.1021/acs.macromol.2c02471)
Supplement: Supplementary file 1 — ma2c02471_si_001.pdf [file ma2c02471_si_001.pdf]

Supporting Information for:

Hydrophilic Aldehyde-functional Polymer Brushes: Synthesis, Characterization and Potential Bioapplications

Emma E. Brotherton,[a] Edwin C. Johnson,[a,*] Mark J. Smallridge,[b] Deborah B. Hammond,[a] Graham J. Leggett,[a] Steven P. Armes[a,*]

a. Dainton Building, Department of Chemistry, The University of Sheffield,

Brook Hill, Sheffield, South Yorkshire, S3 7HF, UK.

b. GEO Specialty Chemicals, Hythe, Southampton, Hampshire SO45 3ZG, UK.

Summary of Contents

Scheme S1. Two-step synthesis of GEO5MA monomer.

Figure S1. Ellipsometry data fits obtained for four PGEO5MA brushes of varying thickness.

Figure S2. High resolution core-line spectra recorded for a BiBB-APTES coated silicon wafer.

Figure S3. XPS survey spectra recorded for an initiator-functionalized silicon wafer, a PGEO5MA brush and the corresponding PAGEO5MA brush.

Figure S4. Absolute and relative change in the ellipsometric dry brush thickness observed for a PGEO5MA brush after exposure to an aqueous solution of 3.0 g dm^{-3} sodium periodate at 22 °C.

Figure S5. Change in the XPS C1s core-line spectrum observed for a PGEO5MA brush (initial dry brush thickness = 50 nm) after exposure to a 3.0 g·dm^{-3} aqueous solution of sodium periodate for up to 120 min at 22 °C.

Figure S6. Reduction in dry brush thickness observed for a PGEO5MA brush (initial dry brush thickness = 41 nm) after exposure to a 65 g·dm^{-3} sodium periodate solution for up to 120 min at 22 °C.

Table S1. Ellipsometry data obtained for six PGEO5MA brushes of varying dry brush thickness and the corresponding periodate-oxidized PGEO5MA brushes.

Figure S7. XPS O1s core-line spectrum for a PGEO5MA brush, periodate-treated PGEO5MA brush and PAGEO5MA brush

Table S2. Summary of the relative amounts of the C-C, C-O and C=O components within the O1s core-line spectra obtained by XPS for the brushes in Figure S7.

Figure S8. C1s, N1s and Si2p core-line spectra recorded as a function of analysis depth for a PHisGEO5MA brush determined during an XPS depth profiling experiment

Figure S9. (a) Raw phase plot and (b) relationship between tracer particle displacement and displacement for a PGEO5MA brush at pH 3 and a PHisGEO5MA brush at pH 4.75.

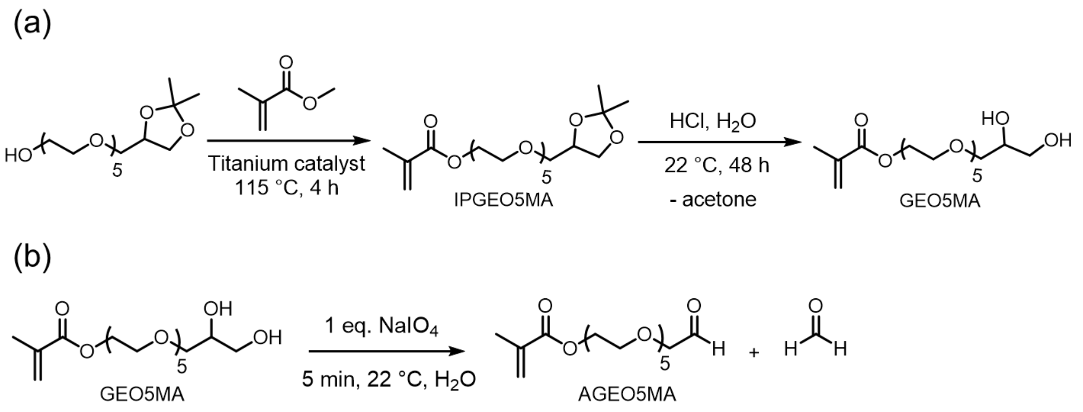

Scheme S2. (a) Two-step synthesis of GEO5MA monomer. A hydroxy-functional isopropylidene glycerol precursor is transesterified with methyl methacrylate to produce IPGEO5MA, before removing the ketal protecting group with acid to afford GEO5MA monomer. (b) Oxidation of GEO5MA in aqueous solution using one equivalent of sodium periodate at 22 °C affords AGEO5MA with formaldehyde as a by-product. The same selective oxidation can be used to convert PGEO5MA homopolymer into PAGEO5MA homopolymer under the same reaction conditions.

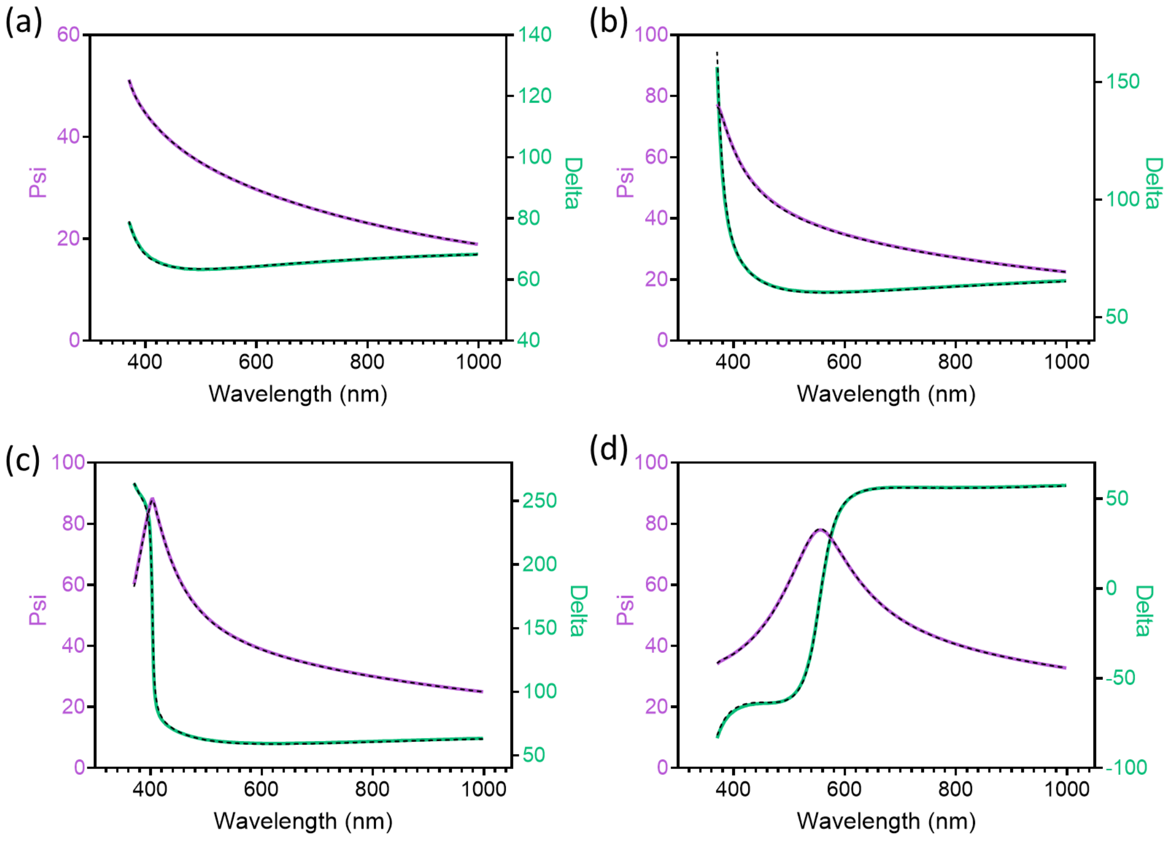

Figure S7. Ellipsometry data (purple and green) and fits (dashed black lines) obtained for four PGEO5MA brushes with the dry brush thicknesses of (a) 66 nm, (b) 74 nm, (c) 84 nm and (d) 120 nm.

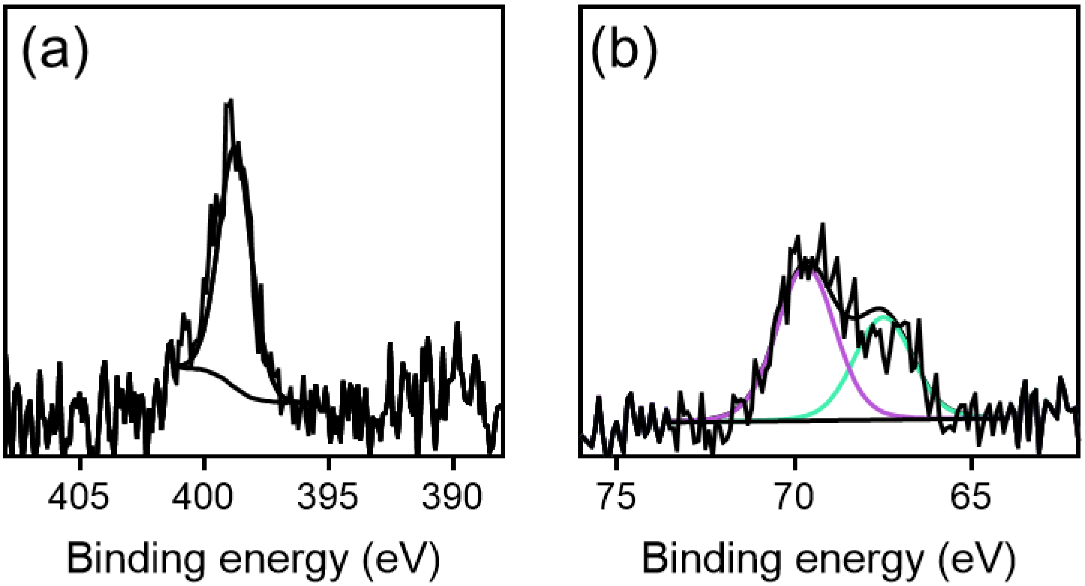

Figure S8. High resolution core-line spectra recorded for a BiBB-APTES coated silicon wafer: (a) N1s and (b) Br3d. In this case, the Br/N atomic ratio is 0.50.

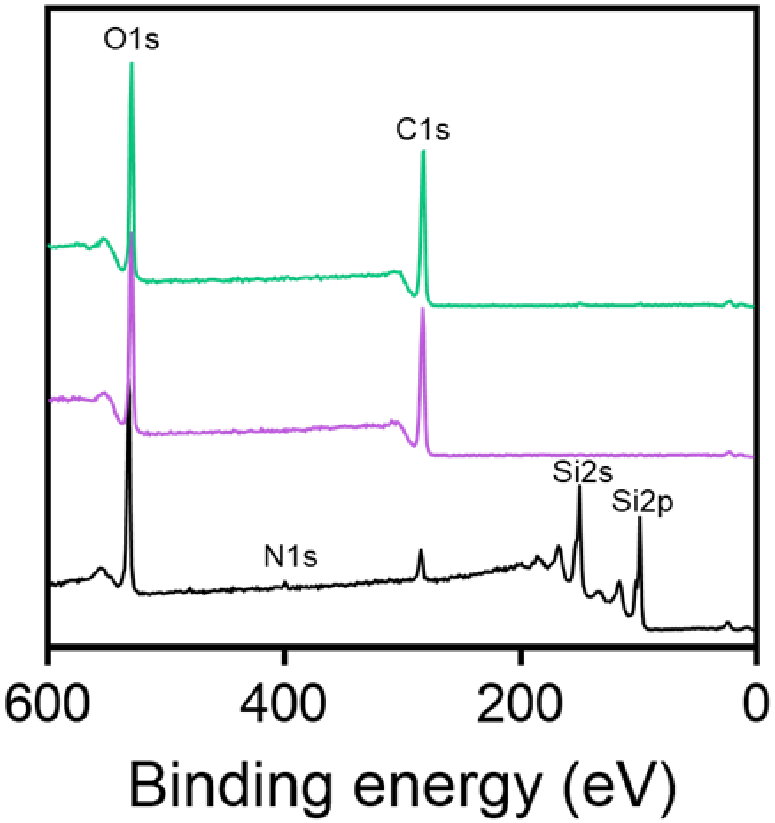

Figure S9. XPS survey spectra recorded for an initiator-functionalized silicon wafer (black spectrum), a PGEO5MA brush (purple spectrum) and the corresponding PAGEO5MA brush (green spectrum). Note the obscuration of the Si2s and Si2p signals arising from the underlying silicon wafer.

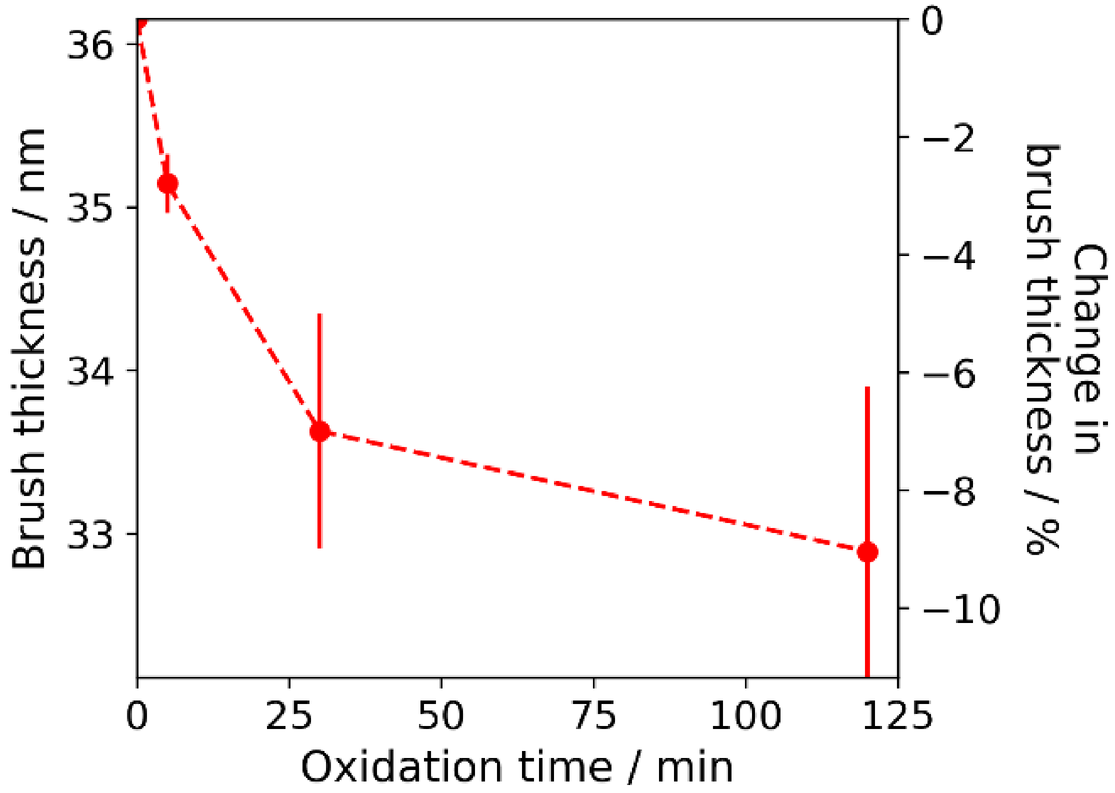

Figure S10. Absolute (left-hand axis) and relative (right-hand axis) change in the ellipsometric dry brush thickness observed for a PGEO5MA brush after exposure to an aqueous solution of 3.0 g dm^{-3} sodium periodate at 22 °C. Under such relatively mild conditions, the change in brush thickness is solely due to selective oxidation (i.e. loss of formaldehyde).

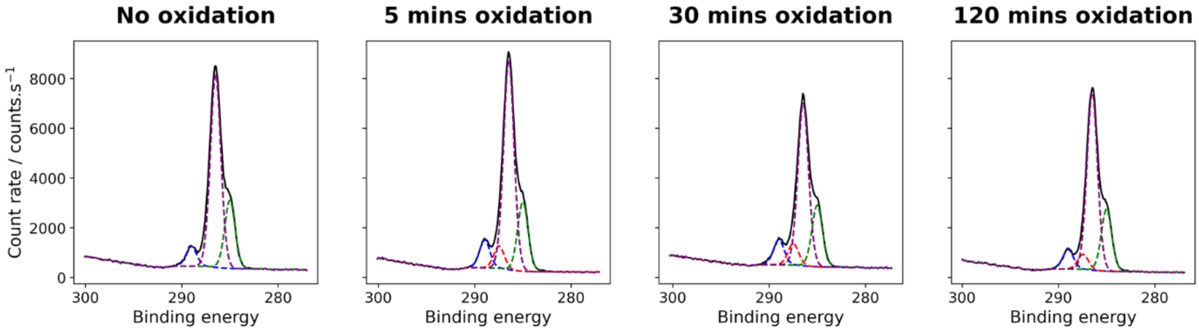

Figure S11. Change in the XPS C1s core-line spectrum observed for a PGEO5MA brush (initial brush thickness = 50 nm) after exposure to a 3.0 g·dm^{-3} aqueous solution of sodium periodate for up to 120 min at 22 °C.

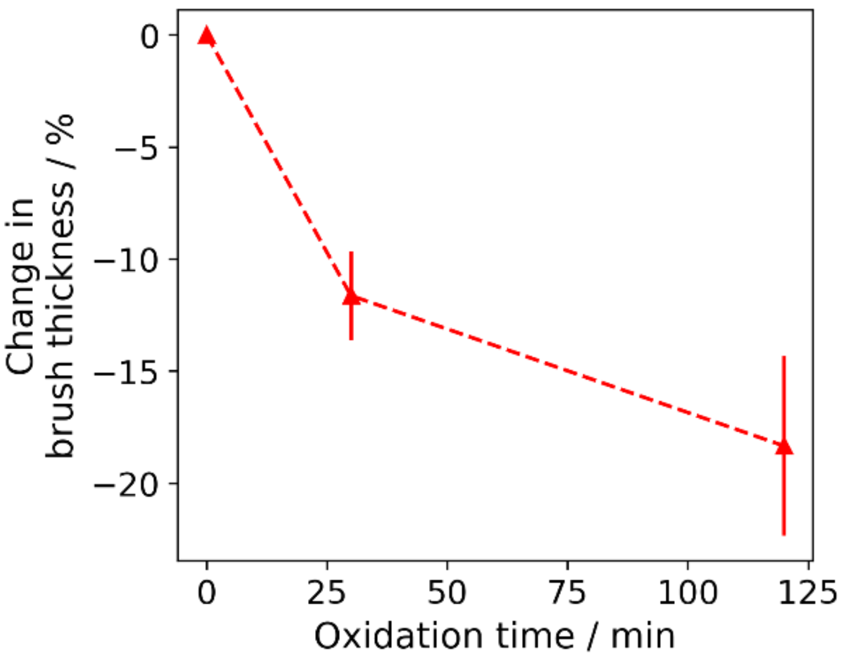

Figure S12. Reduction in dry brush thickness observed for a PGEO5MA brush (initial dry brush thickness = 41 nm) after exposure to a 65 g·dm^{-3} sodium periodate solution for up to 120 min at 22 °C. The initial change in brush thickness observed after 30 min is attributed to the loss of formaldehyde during selective oxidation (complete oxidation should produce an 8.5% mass loss). The subsequent further reduction in brush thickness observed after 2 h indicates chemical degradation and/or partial degrafting of brush chains from the planar silicon wafer at this higher periodate concentration. Brush roughness, evident by deviations in thickness across the surface appears to increase with oxidation time.

Table S2. Ellipsometry data obtained for six PGEO5MA brushes of varying dry brush thickness and the corresponding periodate-oxidized PGEO5MA brushes. The observed reduction in brush thickness is attributed to the loss of one molecule of formaldehyde per GEO5MA repeat unit.

Thickness before oxidation (nm)	Thickness after oxidation (nm)
74	70
84	79
94	84
109	100
118	109
120	114

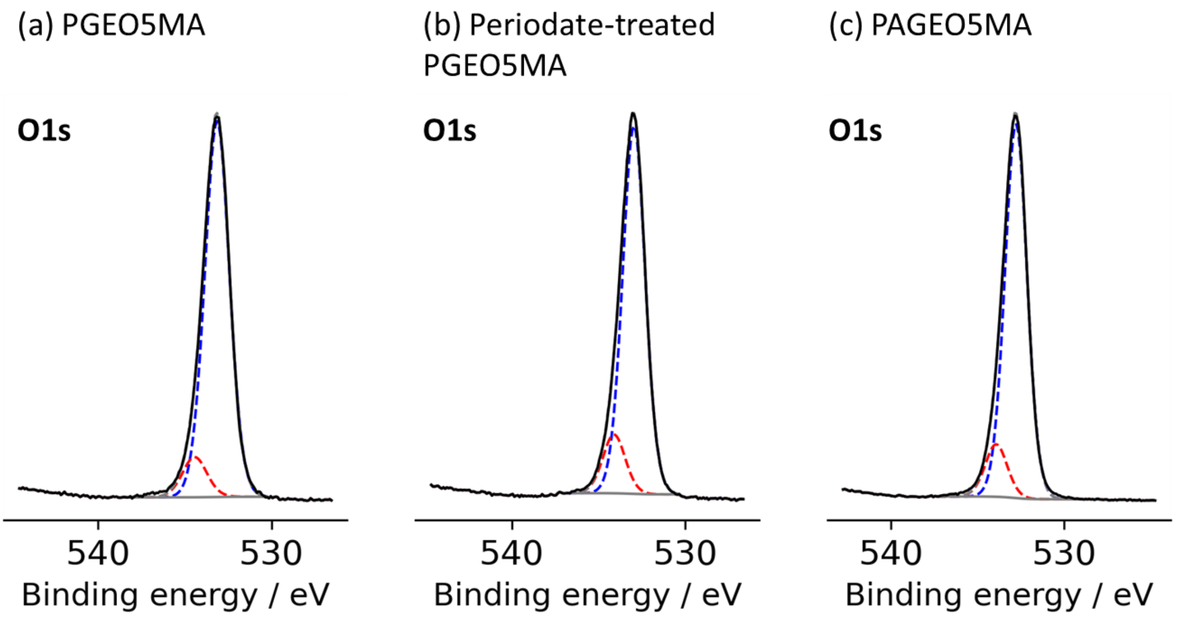

Figure S7. XPS O1s core-line spectrum observed for (a) a PGEO5MA precursor brush (initial brush thickness = 50 nm); (b) after exposure of this PGEO5MA brush to a 3.0 g dm^{-3} aqueous solution of sodium periodate for 30 min at 22 °C; (c) a PAGEO5MA homopolymer brush grown from AGEO5MA monomer. [Solid black lines indicate the raw data. Blue dashed lines correspond to C-O-C, C=O (for the ester group in the case of PGEO5MA and for the ester and aldehyde groups in the case of the periodate-treated PGEO5MA and PAGEO5MA) and C-O-H (for PGEO5MA only). Red dashed lines correspond to C-O-C for the ester group within the methacrylic repeat units for each of these three brushes].

Table S2. Summary of the relative amounts of the C-C, C-O and C=O components within the O1s core-line spectra obtained by XPS analysis of a PGEO5MA brush, a periodate-oxidized PGEO5MA brush and a PAGEO5MA brush (synthesized using AGEO5MA monomer), respectively.

Polymer brush type	Experimental XPS surface composition: atom%		Theoretical XPS surface composition: atom%	
	C-<u>O</u>-C (ether), C=<u>O</u> (ester and/or aldehyde), C-<u>O</u>-H (PGEO5MA only)	C-<u>O</u>-C (ester)	C-<u>O</u>-C (ether), C=<u>O</u> (ester and/or aldehyde), C-<u>O</u>-H (PGEO5MA only)	C-<u>O</u>-C (ester)
PGEO5MA	90.3	9.7	88.9	11.1
Periodate-treated PGEO5MA	86.2	13.8	87.5	12.5
PAGEO5MA	87.7	12.3	87.5	12.5

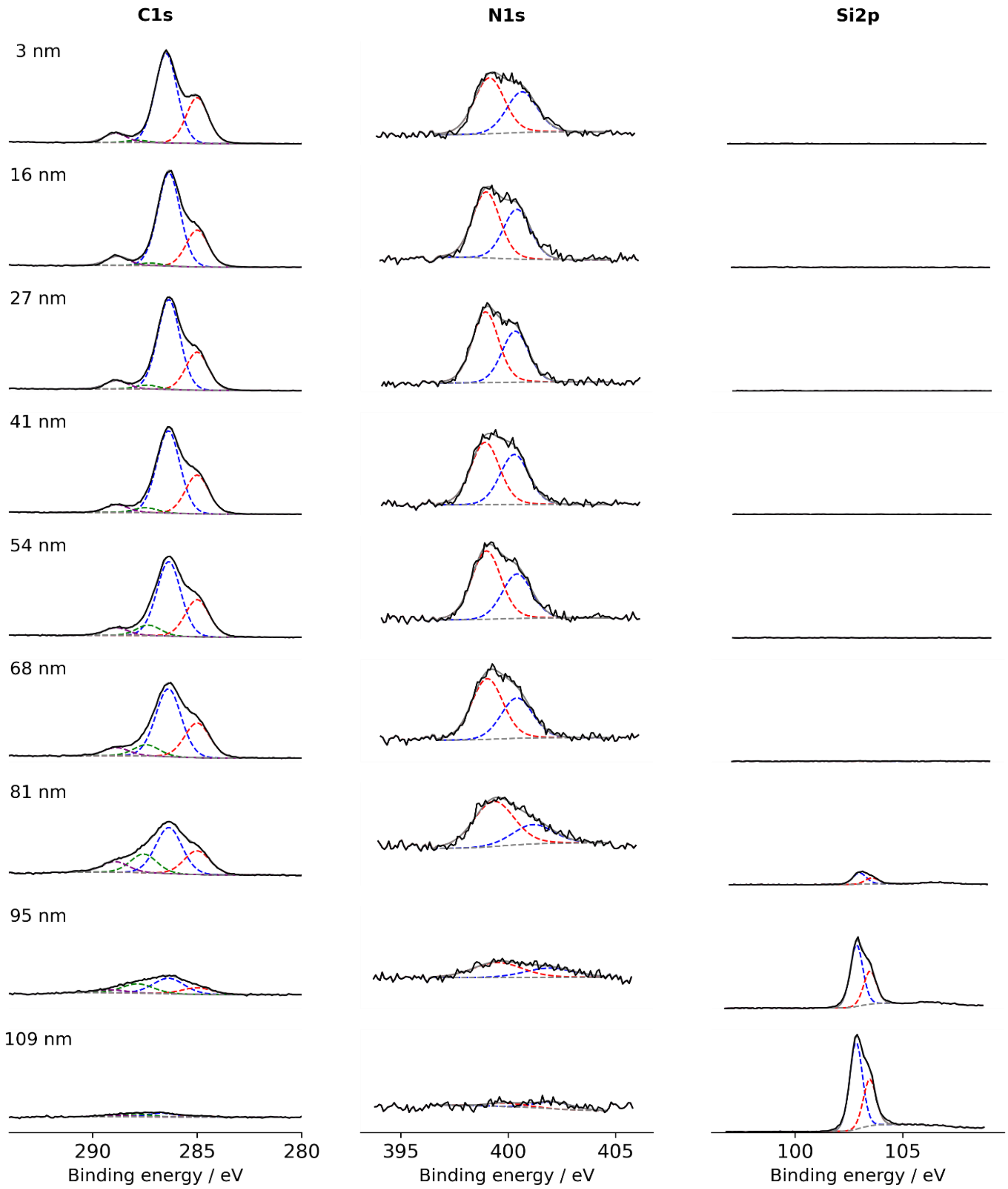

Figure S8. C1s, N1s and Si2p core-line spectra recorded as a function of analysis depth (see C1s spectra) for a PHisGEO5MA brush (dry brush thickness = 109 nm) determined during an XPS depth profiling experiment conducted using an argon cluster source (see main manuscript for further experimental details). The eventual disappearance of the C1s and N1s signals coincide with the appearance of a prominent Si2p signal, which signifies complete etching of the brush layer and exposure of the underlying silicon wafer.

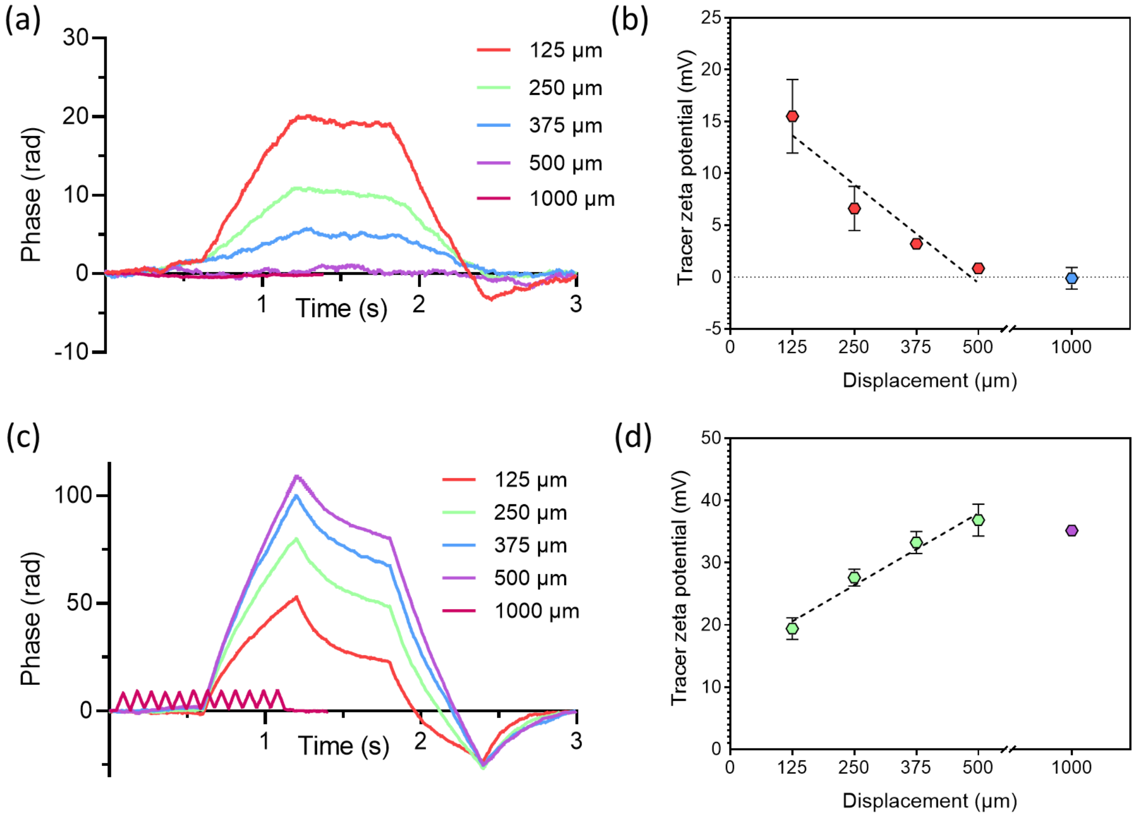

Figure S9. (a) Raw phase plot and (b) relationship between tracer particle displacement and displacement for a PGEO5MA brush (97 nm dry brush thickness) immersed in a 0.003% w/w aqueous dispersion of non-ionic PGMA$_{58}$-PBzMA$_{500}$ tracer particles in 1 mM KCl at pH 3. (c) Raw phase plot and (d) relationship between tracer particle displacement and displacement for a PHisGEO5MA brush (99 nm dry brush thickness) immersed in a 0.003% w/w aqueous dispersion of cationic PMETAC$_{47}$-PBzMA$_{100}$ tracer particles at pH 4.75. [N.B. Slow-field reversal measurements were performed at displacements 125, 250, 375 and 500 µm from the PHisGEO5MA surface. A fast-field reversal measurement was performed at 1000 µm from the PHisGEO5MA surface].
